# Lookism hurts: appearance discrimination and self-rated health in South Korea

**DOI:** 10.1186/s12939-017-0678-8

**Published:** 2017-11-25

**Authors:** Hyemin Lee, Inseo Son, Jaehong Yoon, Seung-Sup Kim

**Affiliations:** 1 Department of Public Health Sciences, Graduate School of Korea University, Anam-ro, Seongbuk-gu, Seoul, South Korea; 2 Asiatic Research Institute, Korea University, Anam-ro, Seongbuk-gu, Seoul, South Korea; 3 Department of Social and Behavioral Sciences, Harvard T.H. Chan School of Public Health, Boston, MA USA

**Keywords:** Appearance discrimination, Lookism, Self-rated health, Emerging adults, South Korea

## Abstract

**Background:**

Despite a growing body of evidence suggesting that discrimination harms health, the association between appearance discrimination and health has been understudied. Our study investigated the association between perceived appearance discrimination and self-rated health among emerging adults using a nationally representative cohort study in South Korea.

**Methods:**

We analyzed the 2nd-10th (2005–2013) waves of cohort data from the Korean Education Employment Panel (KEEP). KEEP consists of two groups of individuals who were 15 (group I) and 18 (group II) years old at the 1st wave of the survey (2004) and were followed annually. Appearance discrimination was assessed at baseline (19 years old: 5th wave for group I, 2nd wave for group II) and at follow-up (24 years old: 10th wave for group I, 7th wave for group II). Responses of appearance discrimination at the two-time points were classified into four groups: 1) never (no discrimination at both baseline and follow-up); 2) repeated (discrimination at both baseline and follow-up); 3) incident (discrimination only at follow-up); and 4) in error (discrimination only at baseline). Multivariate logistic regression was applied to examine the association between reporting patterns of appearance discrimination and poor self-rated health, adjusting for potential confounders.

**Results:**

Compared to those who did not experience appearance discrimination, ‘repeated’ (OR: 3.70; 95% CI: 2.19–6.27) and ‘incident’ (OR: 3.10; 95% CI: 1.99–4.83) groups had a higher odds ratio of poor self-rated health after adjusting for potential confounders including respondents’ body mass index change and baseline self-rated health. However, no significant association was observed among those who reported appearance discrimination ‘in error’.

**Conclusions:**

These results suggest that perceived appearance discrimination is associated with the health of Korean emerging adults considering participants’ reporting patterns of appearance discrimination.

**Electronic supplementary material:**

The online version of this article (10.1186/s12939-017-0678-8) contains supplementary material, which is available to authorized users.

## Background

Sociological literature has accumulated that physical attractiveness is socially constructed, and in general it is positively associated with quality of social experiences [1–3]. Empirical studies show that persons who are not physically attractive are disadvantaged in society, particularly in labor, education, and marriage markets [4–7]. In the labor market, workers whose appearance is socially preferred achieved greater occupational success [4, 5].

While discrimination based on physical attractiveness is prevalent in almost every society [1, 2], it has become a crucial form of discrimination in South Korea (hereafter Korea). Because Korea has experienced relatively compressed industrialization and urbanization during the past decades, education and labor markets as well as social interactions have become highly competitive [8, 9]. Therefore, individual looks, along with gender or age, are often utilized as additional stratifying factors in such institutions.

Several empirical studies investigated the experience of appearance discrimination and its impact on health in Korea [10–12]. According to a study, 24% of 3117 adolescents reported appearance discrimination and those who experienced discrimination due to their appearance were more likely to have suicidal ideation than those who did not [10]. Similar results were observed among Korean adults [11]. And higher prevalence of appearance discrimination was observed among those who have experienced or intend to experience cosmetic surgery than those who did not [12].

According to a report from the *International Society of Aesthetic Plastic Surgery* (2015), Korean people have undergone more than 1,156,000 aesthetic procedures (5.3% of total aesthetic procedures worldwide) [13]. This is the third highest rate of plastic surgery in the world, following countries well-known for cosmetic surgery including the USA and Brazil. When considering population size, Korea has the highest ratio of aesthetic procedures per capita [13]. These statistics imply that excessive concern with physical appearance has emerged as a significant social issue in Korea.

Discrimination is a crucial risk factor that negatively affects one’s health [14, 15]. Although a variety of social distinctions generate discrimination, prior literature has mainly concentrated on the health effects of discrimination based on race/ethnicity, gender, and sexual orientation [14, 15]. Recent studies add other types of discrimination that have been often neglected, such as that due to birth region [16] or age [17].

Among those types of discrimination, lookism or appearance discrimination refers to prejudice or unfair treatment based on one’s physical attractiveness [18]. Studies have examined the health impact of appearance discrimination as one of several types of discrimination [19–21]. One study measured three dimensions of discrimination experiences among immigrant workers—discrimination due to immigrant status, physical appearance, and workplace-related—and examined their associations with health [19]. To our knowledge, however, no research has examined appearance discrimination and its relationship with health using cohort data. In this study, we assessed the prospective association between appearance discrimination and poor self-rated health among emerging adults in Korea.

## Methods

### Data and study population

The Korean Education Employment Panel (KEEP) is a cohort study of 6000 participants from 4175 schools in Korea who have been surveyed annually since 2004. The study consists of 11 annual waves of data (2004–2014) that have been publicly released to date (http://www.krivet.re.kr/ku/ha/kuCAFIn.jsp, accessed 17/07/2017). A three-stage stratified cluster sampling design was adopted with the 1st wave of data. As new samples, 1500 participants from 641 schools were added at 4th wave and 757 participants who graduated from college or 4-year university were included at 7th wave to compensate for those who were taken out of the study. Therefore, the entire KEEP study population totals 8257 participants.

Participants were 15 years old (group I) or 18 years old (group II) at the 1st wave of data. The study population was restricted to emerging adulthood, which represents a period from the late teens through the twenties (18–25 years old) [22] due to the importance of this developmental stage in discrimination experiences [23]. Since we focused on those who are emerging adults, we matched both groups’ age to 19–24 years old using 2nd-10th waves of data. Given the lack of data on appearance discrimination in group I at 4th wave, we could not set the age from 18 years old. Therefore, we used 5th -10th waves for group I and 2nd-7th waves for group II to make a pair of two groups’ age.

### Measures

Appearance discrimination was annually assessed as a lifetime experience using the question, “Have you ever experienced discrimination due to your appearance?” Participants answered “yes” or “no”. Since appearance discrimination measured as lifetime experience, we created a distinct variable for reporting patterns of appearance discrimination using baseline and follow-up data (5th and 10th waves for group I; 2nd and 7th waves for group II). Responses of appearance discrimination at the two-time points were classified into four groups: 1) never (no discrimination at both baseline and follow-up); 2) repeated (discrimination at both baseline and follow-up); 3) incident (discrimination only at follow-up); and 4) in error (discrimination only at baseline). Previous research has considered reports of lifetime discrimination at baseline but not at follow-up as reporting errors [24]. Table 1 shows the labels for reporting patterns of appearance discrimination experiences.

**Table 1:** Labels for reporting patterns of appearance discrimination

Appearance discrimination	Baseline		Follow-up	
	Group I	Group II	Group I	Group II
	5th wave (2008)	2nd wave (2005)	10th wave (2013)	7th wave (2010)
Never	No		No	
Repeated	Yes		Yes	
Incident	No		Yes	
In error	Yes		No	

Prior studies suggest that self-rated health is a reliable variable that reflects overall health status [25]. Self-rated health was annually measured during 2nd-10th waves with single-item Likert scales using the question, “How would you rate your overall health?” Respondents could answer from 1 (very poor) to 5 (excellent), and responses were dichotomized into two categories [1–2: bad health; 3–5: good health].

All potential confounders were measured with self-reported questionnaire at baseline, which was the 5th wave (2008) for group I and 2nd wave (2005) for group II. For demographic variables, sex was distinguished as male or female. Age group was divided into two categories as group I and group II. Residential area at baseline of each group was also included as a categorical variable (i.e. metropolitan area and rural area) in the model. According to previous studies, these socio-demographic variables are the covariates that are potentially confounding the association between discrimination and health [14, 26, 27].

For health-related variables, we selected body mass index (BMI) and baseline self-rated health as covariates since prior empirical studies indicate that respondents’ BMI [28, 29] and self-rated health at baseline [19, 30] were correlated with perceived discrimination as well as health status. BMI was calculated as weight (kg) divided by height squared (m^2^) and was categorized into four groups according to World Health Organization BMI classifications. Since BMI is a time-varying variable, we made a distinct variable to figure out how respondents’ BMI changed between baseline and follow-up. Change of BMI between baseline and follow-up was classified into four categories as follows: 1) no change, 2) toward underweight, 3) toward overweight/obese, and 4) toward normal. Self-rated health at baseline of each group was also adjusted as a potential confounder.

### Data analysis

Multivariate logistic regression was applied to examine the association between reporting patterns of appearance discrimination at baseline and follow-up and self-rated health at follow-up using a cohort study in Korea. Robust standard error was estimated to consider clustering effect among students from the same school by using a school identifier at baseline [31]. All covariates were included in the adjusted model and the association was estimated by odds ratio (OR) and 95% confidence interval (CI). All statistical analyses were conducted using STATA/SE version 13.0 (StataCorp, College Station, TX, USA).

In post-hoc analysis, we examined the association between appearance discrimination and poor self-rated health by controlling for all potential covariates including monthly household income at baseline. Although respondents’ monthly household income may be a confounding factor of the association between appearance discrimination and health, the dataset had missing values in the monthly household income variable. Therefore, we additionally tested the association using several statistical strategies (i.e., complete case analysis, mean substitution analysis, and multiple imputation analysis) to account for missing data, which could otherwise weaken the validity of study results.

Further, we conducted sensitivity analysis by changing the baseline of appearance discrimination in group I and group II. Given that the baselines for group I and group II were the 5th and 2nd wave, we performed four additional analyses of the association between appearance discrimination and poor self-rated health by changing the baseline of group I and group II to 6th wave and 3rd wave, 7th wave and 4th wave, 8th wave and 5th wave, and 9th wave and 6th wave, respectively (Additional file 1: Table S1, S2, S3, S4). We tested whether the association between appearance discrimination and poor self-rated health still exist when the baseline year was closer to the year of follow-up.

## Results

Table 2 shows distribution of the study population and prevalence of poor self-rated health and appearance discrimination by socio-demographic and health-related variables. Participants who were female and in group I were more likely to report poor self-rated health at follow-up. Estimated prevalences of appearance discrimination with four categories of reporting patterns are also suggested in Table 2. Compared to males, female respondents reported more ‘repeated’ and ‘incident’ experiences of appearance discrimination.

**Table 2:** Distribution of study population and prevalence of poor self-rated health at follow-up and reporting patterns of appearance discrimination among emerging adults in Korea, 2005–2013 (*N* = 2,973)

	Distribution	Poor self-rated health^*^		Appearance discrimination^**^				
				Never	Repeated	Incident	In error	
	N (%)	N (%)	*P* ^a^	N (%)	N (%)	N (%)	N (%)	*P* ^b^
Sex			<0.001					<0.001
Male	1,765 (59.4)	87 (4.9)		1,539 (87.2)	26 (1.5)	92 (5.2)	108 (6.1)	
Female	1,208 (40.6)	141 (11.7)		974 (80.6)	48 (4.0)	81 (6.7)	105 (8.7)	
Age			<0.001					0.034
Group I	1,659 (55.8)	159 (9.6)		1,378 (83.1)	49 (3.0)	110 (6.6)	122 (7.4)	
Group II	1,314 (44.2)	69 (5.3)		1,135 (86.4)	25 (1.9)	63 (4.8)	91 (6.9)	
Change of BMI^***^			0.005					0.041
No change	2,348 (79.0)	162 (6.9)		2,003 (85.3)	56 (2.4)	135 (5.8)	154 (6.6)	
Toward underweight	126 (4.2)	18 (14.3)		110 (87.3)	1 (0.8)	3 (2.4)	12 (9.5)	
Toward overweight/obese	199 (6.7)	17 (8.5)		156 (78.4)	8 (4.0)	18 (9.1)	17 (8.5)	
Toward normal	300 (10.1)	31 (10.3)		244 (81.3)	9 (3.0)	17 (5.7)	30 (10.0)	
Residential area			0.461					0.837
Metropolitan area	2,402 (80.8)	180 (7.5)		2,033 (84.6)	62 (2.6)	138 (5.8)	169 (7.0)	
Rural area	571 (19.2)	48 (8.4)		480 (84.1)	12 (2.1)	35 (6.1)	44 (7.7)	
Baseline health status			<0.001					0.038
Good	2,788 (93.8)	185 (6.6)		2,370 (85.0)	67 (2.4)	159 (5.7)	192 (6.9)	
Poor	185 (6.2)	43 (23.2)		143 (77.3)	7 (3.8)	14 (7.6)	21 (11.4)	

^*^Prevalence of poor self-rated health at follow-up

^**^Prevalence of appearance discrimination based on reporting patterns at baseline and follow-up

^***^Change of BMI was classified into four categories according to respondents’ BMI (underweight, normal, overweight, obese) at baseline and follow-up.

1) No change (underweight-underweight, normal-normal, overweight-overweight, obese-obese)

2) Toward underweight (normal-underweight, overweight-underweight, obese-underweight)

3) Toward overweight/obese (normal-overweight, normal-obese, overweight-obese, underweight-overweight, underweight-obese)

4) Toward normal (underweight-normal, overweight-normal, obese-normal, obese-overweight)

^a^ *P*-value of Chi-square test comparing prevalence of poor self-rated health at follow-up across key covariates

^b^ *P*-value of Chi-square test comparing prevalence of appearance discrimination based on reporting patterns at baseline and follow-up across key covariates

We examined the association between the experience of appearance discrimination and poor self-rated health among those who were in emerging adulthood. Table 3 shows the odds ratio of having poor self-rated health among Korean emerging adults according to their reporting patterns of appearance discrimination at baseline and follow-up. Compared to individuals who did not experience any appearance discrimination, higher odds ratio of poor self-rated health was found among ‘repeated’ (OR: 3.70; 95% CI: 2.19–6.27) and ‘incident’ (OR: 3.10; 95% CI: 1.99–4.83) groups. However, no statistically significant association was observed in the ‘in error’ group after adjusting for all potential confounders including BMI change and self-rated health at baseline (OR: 1.21; 95% CI: 0.72–2.02).

**Table 3:** Association between reporting patterns of appearance discrimination and poor self-rated health among emerging adults in Korea, 2005–2013 (*N* = 2,973)

Appearance discrimination	Distribution	Unadjusted		Adjusted^a^	
	N (%)	OR	95% CI	OR	95% CI
Never	2,513 (84.5)	1	Referent	1	Referent
Repeated	74 (2.5)	4.79^*^	2.85-8.05	3.70^*^	2.19–6.27
Incident	173 (5.8)	3.38^*^	2.17-5.26	3.10^*^	1.99-4.83
In error	213 (7.2)	1.54	0.96-2.47	1.21	0.72-2.02

^*^ *P* < 0.001

^a^Adjusted for sex, age, change of BMI at baseline and follow-up, residential area, and baseline self-rated health

To control for the monthly household income variable which has missing values, we examined the association between appearance discrimination and poor self-rated health using complete case analysis, mean substitution analysis, and multiple imputation analysis. Complete case analysis examined the association only with the available data of monthly household income, while mean substitution analysis replaced missing values of monthly household income with sample mean. In multiple imputation analysis, a higher odds ratio of having poor self-reported health was found among ‘repeated’ (OR: 3.70; 95% CI: 2.19–6.27) and ‘incident’ (OR: 3.10; 95% CI: 1.99–4.83) groups compared to those who never reported appearance discrimination at baseline and follow-up. No statistically significant association was observed in the ‘in error’ group. In all three analyses, we obtained consistent results (Table is not shown).

**Fig. 1:**
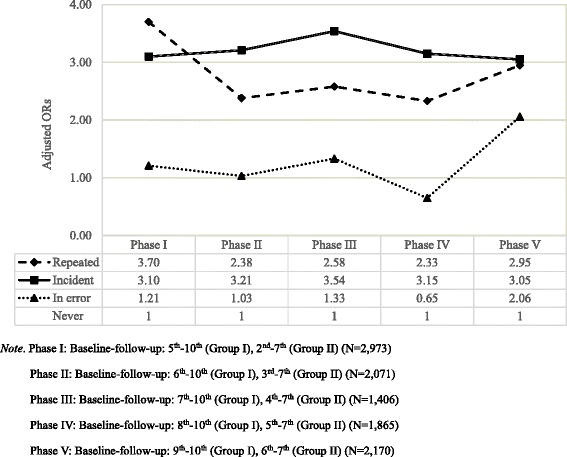
Sensitivity analysis of the association between appearance discrimination and poor self-rated health with changing the baseline waves of appearance discrimination as an independent variable

Furthermore, sensitivity analysis was conducted to assess the robustness of findings by changing the baseline year of appearance discrimination as an independent variable in both groups. Results were consistent with the primary results in Table 3 that those who experienced ‘repeated’ or ‘incident’ appearance discrimination had a higher odds ratio of having poor self-reported health in comparison to those in the ‘never’ group (Fig. 1, Additional file 1: Table S1, S2, S3, S4).

## Discussion

A growing body of evidence supported that perceived discrimination is associated with various negative health outcomes [14, 15]. However, little research investigated the impact of appearance discrimination on health using a cohort study. We analyzed the prospective association between appearance discrimination and self-rated health among Korean emerging adults using a nationally representative cohort data. Along with previous studies [24, 32], this research found that ‘repeated’ or ‘incident’ experience of appearance discrimination is statistically significantly associated with poor self-rated health among Korean emerging adults.

Discrimination experiences in a certain period of life, especially in an early stage, can impinge on one’s life [23, 33]. Those who are in emerging adulthood are undergoing critical demographic transitions, such as finishing school, entering the labor market, and beginning to explore their identity [22]. In the transition process, these individuals are considered as socially disadvantaged and thus they are more vulnerable to the effects of discrimination than people in other life stages [23, 33]. A recent study suggests that those who are in the period of adolescence through emerging adulthood with persistent discrimination experiences are more likely to engage in risky health behaviors (i.e., substance or alcohol use) [23, 33]. That is why it is particularly salient to examine the association between perceived discrimination and negative health conditions among individuals in this developmental stage.

Although physical attractiveness has no consensus definition, there is a universal idea of physical attractiveness irrespective of cultural background [34, 35]. Individuals’ appearance is one of the visible characteristics, and it includes weight, height, and other aspects including makeup, hair styles in general [2]. For instance, skin color is regarded as an appearance characteristic in countries that are multiracial/ethnic. As the number of immigrants has increased in Korea, discrimination against immigrants is pervasive [27]. Therefore, skin color should be considered as an element of appearance when interpreting the results of this study.

Prior studies stressed traditional sex roles in the perception of beauty [2, 3], which means that women are assumed to be more affected by their looks than men. However, we did not find significant gender differences in the association between reported appearance discrimination and poor self-rated health among emerging adults in Korea. Our findings correspond with several studies that concluded both men and women are under similar influences of the social distinction of looks [36–38]. Nonetheless, future research needs to explore how the gendered mechanism of appearance discrimination affects women and men differently. There could be a chance that women as a socially disadvantaged group underreport their discrimination experiences [39]. Gender differences in reporting discrimination experience and the mechanisms that how the experience of appearance discrimination influences on health should be considered in future research.

Our research has some limitations. First, appearance discrimination as an independent variable was annually measured with a single-item question, therefore, it is difficult to capture various elements of discrimination including domain, strength, and perpetrator. Standardized questionnaires [40, 41] should be used to more elaborately assess respondents’ discrimination experiences. Second, this study cannot rule out the possibility that there could be residual confounders which were not properly applied in the analysis. Previous studies on appearance discrimination added educational level, occupation, and marital status as potential confounders in the model besides age, gender, BMI, income [10, 11, 19]. However, we did not include educational level, employment status, and marital status as covariates since the difference is relatively small in our study population. Given respondents’ age as early and mid-20s, most of them are college or university students and few respondents are married (less than 3.5% of respondents are married at follow-up of each age group).

This study also has key strengths. Compared to prior studies, we investigated the prospective association between reporting patterns of appearance discrimination and poor self-rated health. Previous literature underscored the need for future research to examine cohort studies to draw causal conclusions about the relationship between perceived discrimination and health [14]. There are a few studies using longitudinal data to analyze the harmful health impact of discrimination including work-related discrimination [42] and racial discrimination [24, 43]. However, little research has been conducted regarding appearance discrimination and health.

## Conclusions

This study found the association between appearance discrimination and health after adjusting for potential confounders including change of respondents’ BMI and baseline self-rated health. These results were consistent with sensitivity analysis considering imputed monthly household income variable in the model. We also tested the robustness of our findings with changing the baseline year of appearance discrimination.

To the best of our knowledge, this is the first research to examine the prospective association between appearance discrimination and self-rated health among emerging adults in Korea. Further, this study demonstrated that the impact of discrimination experiences, especially due to physical appearance, on poor self-rated health must be understood in the context of individuals’ life course. Additional studies are now needed to analyze the effect of appearance discrimination on more various health outcomes and among diverse study populations.

## Additional file

Additional file 1:
**Table S1.** Association between experience of appearance discrimination and poor self-rated health among emerging adults in Korea (Baseline-follow-up: 6th-10th for Group I, 3rd-7th for Group II; *N* = 2,071). **Table S2.** Association between experience of appearance discrimination and poor self-rated health among. emerging adults in Korea (Baseline-follow-up: 7th-10th for Group I, 4th-7th for Group II; *N* = 1,406). **Table S3.** Association between experience of appearance discrimination and poor self-rated health among emerging adults in Korea (Baseline-follow-up: 8th-10th for Group I, 5th-7th for Group II; *N* = 1,865). **Table S4.** Association between experience of appearance discrimination and poor self-rated health among emerging adults in Korea (Baseline-follow-up: 9th-10th for Group I, 6th-7th for Group II; *N* = 2,170). (DOCX 20 kb)

## Abbreviations

BMI: Body Mass Index
KEEP: Korean Education Employment Panel
