# Supplementary material for: Lookism hurts: appearance discrimination and self-rated health in South Korea
Source: Int J Equity Health. 2017 Nov 25;16:204. doi: 10.1186/s12939-017-0678-8 (PMC5702199; doi:10.1186/s12939-017-0678-8)
Supplement: Additional file 1: — Table S1. Association between experience of appearance discrimination and poor self-rated health among emerging adults in Korea (Baseline-follow-up: 6th-10th for Group I, 3rd-7th for Group II; N = 2,071). Table S2. Association between experience of appearance discrimination and poor self-rated health among. emerging adults in Korea (Baseline-follow-up: 7th-10th for Group I, 4th-7th for Group II; N = 1,406). Table S3. Association between experience of appearance discrimination and poor self-rated health among emerging adults in Korea (Baseline-follow-up: 8th-10th for Group I, 5th-7th for Group II; N = 1,865). Table S4. Association between experience of appearance discrimination and poor self-rated health among emerging adults in Korea (Baseline-follow-up: 9th-10th for Group I, 6th-7th for Group II; N = 2,170). (DOCX 20 kb) [file 12939_2017_678_MOESM1_ESM.docx]

Additional file 1: Table S1 Association between experience of appearance discrimination and poor self-rated health among emerging adults in Korea (N=2,071)

| Appearance discrimination^†^ | Distribution |  | Unadjusted | | Adjusted^a^ | |
| --- | --- | --- | --- | --- | --- | --- |
|  | N (%) |  | OR | 95% CI | OR | 95% CI |
| Never | 1,707 (82.4) |  | 1 | Referent | 1 | Referent |
| Repeated | 80 (3.9) |  | 2.62^*^ | 1.44-4.78 | 2.38^*^ | 1.28–4.44 |
| Incident | 111 (5.4) |  | 3.48^**^ | 2.22-5.46 | 3.21^**^ | 2.03-5.08 |
| In error | 173 (8.4) |  | 1.32 | 0.81-2.16 | 1.03 | 0.61-1.77 |

^*^*P* < 0.01, ^**^ *P* < 0.001

^a^Adjusted for sex, age, change of BMI at baseline and follow-up, residential area, and baseline self-rated health.

^†^Baseline and follow-up waves: 6^th^-10^th^ (Group I), 3^th^-7^th^ (Group II)

Additional file 1: Table S2 Association between experience of appearance discrimination and poor self-rated health among emerging adults in Korea (N=1,406)

| Appearance discrimination^†^ | Distribution |  | Unadjusted | | Adjusted^a^ | |
| --- | --- | --- | --- | --- | --- | --- |
|  | N (%) |  | OR | 95% CI | OR | 95% CI |
| Never | 1,157 (82.3) |  | 1 | Referent | 1 | Referent |
| Repeated | 62 (4.4) |  | 3.45^**^ | 1.85-6.43 | 2.58^*^ | 1.27–5.22 |
| Incident | 78 (5.6) |  | 3.65^**^ | 2.06-6.47 | 3.54^**^ | 1.91-6.54 |
| In error | 109 (7.8) |  | 1.58 | 0.88-2.84 | 1.33 | 0.72-2.46 |

^*^*P* < 0.01, ^**^ *P* < 0.001

^a^Adjusted for sex, age, change of BMI at baseline and follow-up, residential area, and baseline self-rated health.

^†^Baseline and follow-up waves: 7^th^-10^th^ (Group I), 4^th^-7^th^ (Group II)

Additional file 1: Table S3 Association between experience of appearance discrimination and poor self-rated health among emerging adults in Korea (N=1,865)

| Appearance discrimination^†^ | Distribution |  | Unadjusted | | Adjusted^a^ | |
| --- | --- | --- | --- | --- | --- | --- |
|  | N (%) |  | OR | 95% CI | OR | 95% CI |
| Never | 1,611 (86.4) |  | 1 | Referent | 1 | Referent |
| Repeated | 76 (4.1) |  | 2.97^**^ | 1.63-5.38 | 2.33^*^ | 1.25–4.34 |
| Incident | 93 (5.0) |  | 3.15^**^ | 1.74-5.72 | 3.15^*^ | 1.63-6.10 |
| In error | 85 (4.6) |  | 0.82 | 0.33-2.06 | 0.65 | 0.24-1.76 |

^*^*P* < 0.01, ^**^ *P* < 0.001

^a^Adjusted for sex, age, change of BMI at baseline and follow-up, residential area, and baseline self-rated health.

^†^Baseline and follow-up waves: 8^th^-10^th^ (Group I), 5^th^-7^th^ (Group II)

Additional file 1: Table S4 Association between experience of appearance discrimination and poor self-rated health among emerging adults in Korea (N=2,170)

| Appearance discrimination^†^ | Distribution |  | Unadjusted | | Adjusted^a^ | |
| --- | --- | --- | --- | --- | --- | --- |
|  | N (%) |  | OR | 95% CI | OR | 95% CI |
| Never | 1,866 (86.0) |  | 1 | Referent | 1 | Referent |
| Repeated | 87 (4.0) |  | 4.29^**^ | 2.73-6.74 | 2.95^**^ | 1.72-5.07 |
| Incident | 104 (4.8) |  | 2.99^**^ | 1.91-4.69 | 3.05^**^ | 1.72-5.41 |
| In error | 113 (5.2) |  | 3.11^**^ | 1.94-5.01 | 2.06^*^ | 1.03-4.09 |

^*^*P* < 0.05, ^**^ *P* < 0.001

^a^Adjusted for sex, age, change of BMI at baseline and follow-up, residential area, and baseline self-rated health.

^†^Baseline and follow-up waves: 9^th^-10^th^ (Group I), 6^th^-7^th^ (Group II)
